# Influence of Yarn and Fabric Properties on Mechanical Behavior of Polymer Materials and Its Retention over Time

**DOI:** 10.3390/polym16121725

**Published:** 2024-06-17

**Authors:** Ivana Salopek Čubrić, Goran Čubrić

**Affiliations:** 1 Department of Textile Design and Management, University of Zagreb Faculty of Textile Technology, Prilaz baruna Filipovića 28 a, 10000 Zagreb, Croatia; 2 Department of Clothing Technology, University of Zagreb Faculty of Textile Technology, Prilaz baruna Filipovića 28 a, 10000 Zagreb, Croatia; goran.cubric@ttf.unizg.hr

**Keywords:** yarn, polymer, mechanical properties, polyester, recycled, material, aging, measurement, surface roughness, abrasion, tensile property, compression

## Abstract

The mechanical properties of textile materials play a crucial role in determining their comfort, functionality, performance, safety, and aesthetics. Understanding and optimizing these properties is essential to meet consumer demands. Key aspects of mechanical properties, such as surface roughness, abrasion resistance, and compression, have a significant impact on the touch and durability of the material, as demonstrated by various research studies. This study focuses on analyzing the mechanical properties of materials produced of different polymer yarns and their changes under combined aging factors. The findings emphasize the significance of textile abrasion resistance and surface roughness measurement, particularly for aged materials. Although the use of recycled polyester yarn is sustainable and offers advantages such as higher tensile strength, the results have shown that the use of conventional polyester yarn is more advantageous overall as it has higher abrasion resistance, a smoother surface texture, and better elasticity retention after aging. The insights presented are vital for designing high-performance sportswear, which is crucial in today’s competitive environment.

## 1. Introduction

The mechanical properties of textile materials play a fundamental role in determining the functionality, comfort, safety, and visual appeal of textile products for different purposes. Understanding and optimizing these properties are essential to meet the different needs and preferences of consumers. A large number of distinct physiological and psychological reactions of the human body, in combination with the mechanical properties of textile materials, create a subjective perception of material that can change over time. Material feel can be a multidimensional concept that encompasses some material properties, including the material’s surface roughness, abrasion resistance, compression, and tensile properties [1,2].

The surface roughness of textile materials refers to height differences in the high-frequency range of variations on the material surface and can be either subjectively assessed or instrumentally tested. The studies that focused on the perception of roughness by recruited evaluators showed that among a variety of material properties (including material thickness, elasticity, and softness), surface roughness is best recognized and appreciated by the evaluators of different genders and age ranges [3,4]. Assuming that evaluators represent a good sample of textile consumers, the above data suggest that the characteristic of roughness is perceived as more important than other material touch attributes when a product is purchased. This is the main reason why the property of material roughness should be given special consideration when comparing similar textile materials and when studying the behavior of the material during long-term use. Chae et al. [5] investigated the influence of surface roughness on the color effect of a series of cotton fabrics. The authors showed that a difference in the surface roughness of the material of more than 0.43 mm can lead to differences in the color appearance of the product, which in turn can lead to a negative perception by the consumer or wearer of the product. The study conducted by Lee [6], which focused on the influence of different patterns on the roughness of 100% polyester knitted fabrics, indicated higher values for tuck, purl, and plain jersey stitch patterns. As far as the influence of aging on the changes in polymer materials is concerned, the measurements on the FTT tester have shown that the properties of standard and artificially aged polyester fabric (aging duration 85 h) differ significantly. In this case, the smoothness rating (as the second part of the roughness–smoothness attribute pair) of the artificially aged polyester fabric drops to 2.0, compared to the standard polyester fabric with 5.0 [7]. 

The abrasion resistance of materials is an important factor in determining the durability and long-term performance of a textile product. It refers to the wear simulation that leads to the deterioration of fibers and yarns due to mechanical friction between surfaces. Therefore, its evaluation is crucial for manufacturers to ensure product quality and performance during prolonged use under different conditions. So far, the studies have investigated the effects of various factors on the abrasion resistance of materials. The studies have mainly focused on investigating materials made from natural fibers or comparing natural and synthetic fibers. Mikučioniene et al. [8] investigated the abrasion resistance of knitted fabrics made of natural and peat fibers in different compositions. The results of tests on the Martindale abrasion tester showed that all designed knitted fabrics had a high abrasion resistance. The authors conclude that all could be used for clothing manufacture, as their surface was not broken even after 50,000 abrasion cycles. Kilic et al. [9] focused on knitted fabrics made of cotton, polyester, acrylic, and modal fibers. The results of the abrasion tests, which were also carried out using the Martindale abrasion tester, showed that knitted fabrics made of polyester have the lowest mass loss, which the authors explain with the higher tenacity of polyester fibers. The abrasion of cotton knitted fabrics with the addition of 1% and 3% of elastane showed that knitted fabrics with a lower percentage of elastane had a better visual appearance after abrasion but experienced the thinning at a lower number of abrasion cycles [10]. The materials of cotton, wool, and cotton/polyester yarns were the focus of the study conducted by Gupta et al. [11]. The tests showed that the knot density of 6 inch^−1^ resulted in the lowest abrasion loss. In terms of environmental factors and specific conditions of use (like care process, UV radiation, high/low temperatures, contact with pool or seawater, etc.), these factors have been shown to accelerate material degradation, which over time can be critical to the future use of a product. Jabbar et al. [12] focused on the abrasion of fabrics made from cotton yarns during ordinary use and washing, as well as the effects on fiber damage, i.e., the generation and release of fragmented fibers. The study showed that the specimen produced by conventional cotton yarns releases a greater amount of fragmented fibers than compact and SIRO cotton yarns.

The property of material compression refers to the reduction in thickness or volume when the material is subjected to external pressure or force. It has been a subject of previous investigations, connecting this property with the final use of the product [13,14]. The compression of certain products, such as sports or medical stockings, is gaining in importance due to the targeted treatment of chronic venous insufficiency [15]. In knitted materials, the compression behavior can be influenced by the type and construction of the yarn, the structural properties of the fabric, or the knitting technique used. Changing just one of these parameters can lead to materials with significantly different compressibility. With regard to the parameters of the knitting process, which primarily influence the arrangement of the loops and the tightness of the structural elements, knitted fabrics can exhibit different longitudinal and transverse compressibility. Whether the aim is to increase the comfort of sportswear, improve blood circulation in the body, or support the muscles, the compression properties of textile materials play an important role in their functionality and performance. 

A number of researchers have investigated the tensile properties of textile materials using both bursting and tensile tests [16,17,18]. The use of bursting tests for knitted fabrics may be more accurate, but the use of tensile tests gives an interesting insight into the different behavior of knitted material, especially when samples are carefully prepared to be tested in the directions of wales/courses. Studies that have used different testing methods showed that tensile properties depend on the composition of the material, the manufacturing method, the type of polymer used, and other parameters [19,20].

During their life cycle, polymer materials are regularly subjected to an aging process that impairs their performance. Recent researchers have focused on understanding these changes, which has also led to the development of various test methods to accelerate material aging. The degradation of polymers, particularly polyesters, is a growing concern due to accumulation in the environment. Studies have shown that exposure to various environmental conditions affects mechanical and physical properties, including molecular degradation and crystalline phase change [21,22,23]. Changes in material properties can affect the perception of comfort, which is crucial for apparel products. While interest in sports-related materials is increasing, the literature review pointed out that specific aging protocols for functional sportswear are lacking. Previous studies have shown that aging has an effect on certain physical–mechanical characteristics, but it is concluded that these changes should not significantly affect the athlete’s performance [24,25,26,27]. 

The global consumption of fibers and materials for the production of sportswear and sporting goods has increased significantly over the last decade. The growing interest in purchasing sportswear is the result of numerous social factors including the increased concern for personal health and well-being, as well as the promotion of sports activities on a global scale. Polyester is the most commonly used fiber in the production of sportswear, as it is valued for several properties (for example, it is durable, elastic, dries quickly, and often does not require ironing). The downside is the fact that its production has a negative impact on the environment. Polyester recycling is a sustainable option, i.e., a way to continue to benefit from the properties of polyester without harming the environment. However, this raises the question of whether the properties that characterize conventional polyester will be retained for recycled polyester and, more so, how these properties change over time. As far as the authors are aware, there is still a lack of studies looking at the aging of such materials in a specific sports training environment. Therefore, the study presented in this paper focuses on investigating the mechanical properties of polyester materials used for sportswear, namely conventional polyester, recycled polyester, and conventional polyester with added elastane. In order to observe the changes in material properties through use, the materials were aged using an aging protocol containing combined aging factors representative of the sport, in this case, football, and the changes in material properties through aging are discussed. The intention is to provide an insight into how different polyester materials change over time in terms of their mechanical properties so that the concluding remarks can be taken into account when developing or purchasing a product for the same purpose, i.e., the manufacture of sportswear.

## 2. Materials and Methods

### 2.1. Materials

Polymer materials consisting of conventional polyester yarn (both non-plated and plated with elastane) as well as recycled polyester yarn were selected for this study. All materials were produced as knitted structures and represented the typical material for the production of sportswear used in professional football. The mass per unit area of selected materials is 132 ± 2 g/m^2^. The polymer materials were exposed to outdoor weathering during the summer season in a moderate European continental climate with an average air temperature of 30.91 °C, a UV index of 6, and an average humidity of 50%. Parallel to the outdoor exposure of the polymer materials, a dilution of acid sweat in powder form with a pH value of 5.5 was added to the samples to simulate the influence of the player’s intensive sweating during training. The duration of weathering corresponded to 8 weeks of intensive training for a professional football player. After each simulated training session, the material was washed in a washing machine with a detergent containing no optical bleach or phosphates. The washing temperature was set to 30 °C, and the materials were then air-dried. The details of the materials with the corresponding designations can be found in Table 1.

### 2.2. Methods of Measurement 

**Figure 1:**
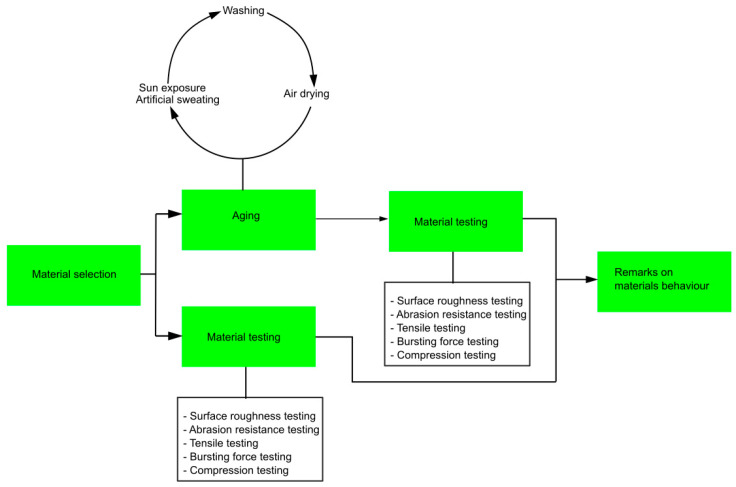
Schematic view of the methodology.

The schematic view of the methodology used within this study is given in Figure 1.

The evaluation of material properties before and after aging included the following mechanical properties: surface roughness, abrasion resistance, tensile properties, bursting force, and compression.

#### 2.2.1. Surface Roughness Testing

For the testing of polymer material surface roughness, the roughness tester PCE-RT 2000 (PCE Instruments UK Ltd., Southampton, UK) was used. The testing is conducted according to the principles defined in international standard ISO 3274 [28]. 

For analyzing surface structure and interpreting the results, two roughness parameters were used:

1. Mean height of profile elements (Rc), and
2. Arithmetical mean deviation of assessed profile (Ra).

In order to define the mean height of profile elements (Rc), all profile elements that consist of one peak and its adjacent valley need to be observed. The parameter mean height of profile elements is calculated using the distance between peak and valley (Zt), i.e., as the following:

(1) $$Rc=\frac{1}{m}\sum_{i=1}^{m}Zt_{i}$$

where m—number of elements in the sampling length, Zt_i_—height of the profile element.

**Figure 2:**
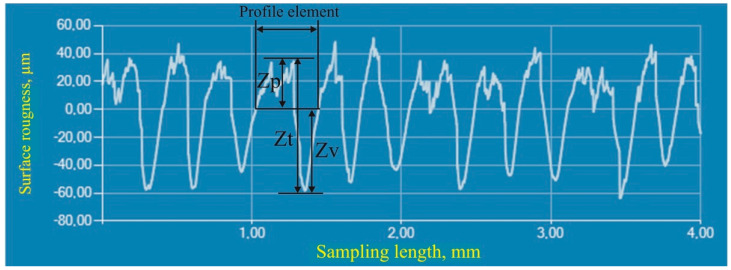
The profile element with the highest point of the profile peak (Zp), the distance between peak and valley (Zt), and the deepest point of the profile valley (Zv).

The distance between the peak and valley (Zt) for each element adds up the distance between the mean line and the highest point of the profile peak (Zp) and the distance between the mean line and the deepest point of the profile valley (Zv), Figure 2. 

The maximum Zp in the sampling length is called the maximum profile peak height (Rp), while the maximum Zv is called the maximum profile valley depth (Rv). 

The second parameter, arithmetical mean deviation of assessed profile (Ra) represents the average value of the absolute values of the profile variations Z_1_, Z_2_, …, Z_n_) It is calculated using the following equations:

(2) $$Ra=\frac{\left|Z_{1}\right|+\left|Z_{2}\right|+\ldots +\left|Z_{n}\right|}{n}$$

(3) $$Ra=\frac{1}{n}\sum_{i=1}^{n}\left|Z_{i}\right|$$

(4) $$Ra=\frac{1}{lr}\int_{0}^{lr}\left|Z(x)\right|dx$$

**Figure 3:**
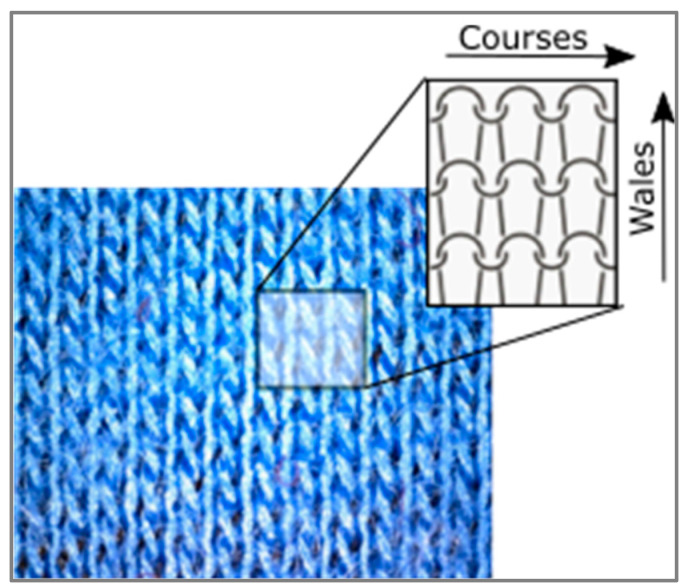
The structure of knitted material with defined directions of courses and wales.

During the testing, the roughness tester was placed on a flat surface in the ambient with stabile standard conditions (temperature of 20 ± 2 °C, relative humidity of 65 ± 3%, and air velocity of 0.15 ± 0.05 m/s). The measurement was carried out on the face and back of the material, in both directions of courses and wales (Figure 3). 

#### 2.2.2. Abrasion Resistance Testing

The abrasion resistance of the material was assessed using the AquAbrasion Tester (James Heal, Halifax, UK). This special device works according to the Martindale principle and was developed to evaluate the durability of materials under various environmental conditions. For the test, the samples were cut to a diameter of 38 ± 5 mm. Each sample was clamped in the tester’s sample holder. In order to ensure the stability of the material within the holder, two layers of each specimen were inserted and clamped. For the abrasion of materials, a standard woven fabric was placed on the abrasion basis of the instrument. The polymer materials were subjected to a load of 9 kPa, rubbing it against an abrasive medium in a movement following the Lissajous curve. The mass loss method was used to analyze the abrasion resistance of the material. In this method, the samples were weighed before abrasion and after a certain number of abrasion cycles. The following numbers of cycles were defined for the test: 1000, 2500, 3500, and 5000.

#### 2.2.3. Tensile Testing

The tensile properties of the materials, including force at break and elongation at break, were examined following the protocol outlined in the international standard ISO 13934-1 [29]. For the testing, the Statimat M tensile tester (Textechno, Mönchengladbach, Germany) was used. For the purpose of testing, samples in the dimensions of 50 × 200 mm that were conditioned in a standard test atmosphere at a temperature of 20 ± 2 °C and relative humidity of 65 ± 3% were used. During the testing, the distance between the clamps of the tensile tester was set at 100 mm.

#### 2.2.4. Bursting Force Testing

In order to compare the results of the tensile force action on a strip specimen of knitted fabric with the action of force when bursting a material, the bursting force testing was conducted. This testing method is independent of the specimen’s cutting direction, as bursting occurs naturally in the direction of lowest resistance. For testing, a burst tester (Apparecchi Branca, Milan, Italy) featuring a polished steel ball with a diameter of 25.40 ± 0.005 mm was used. The testing was conducted according to standard ASTM D3787 [30]. For each of the six knitted materials investigated, five circular specimens with a diameter of 50 ± 1 mm were tested, and the value of bursting force was calculated as an average value.

#### 2.2.5. Compression Testing

**Figure 4:**
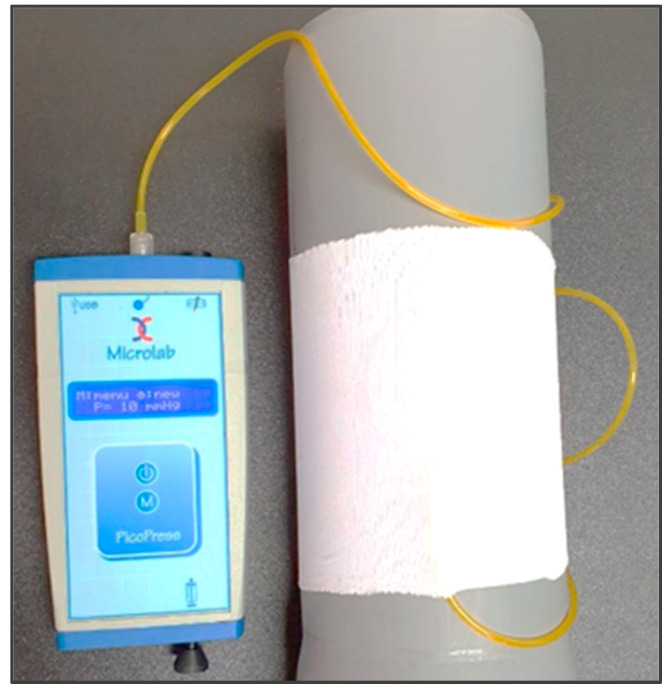
Positioning of the material over a cylinder and connection to the PicoPress apparatus.

The material compression was assessed using a PicoPress apparatus (Microlab electronica s.a.s., Bergamo, Italy) and a cylinder with a specific circumference. This device has a measurement range of 0 to 189 mmHg, a measurement precision of ±3 mm Hg, and a maximum pressure capacity of 300 mm Hg. Before the start of the measurement, the material was cut and tailored to correspond to the dimensions of the cylinder. The material specimen was stretched over a cylinder of a particular diameter (240, 350, or 395 mm), below which a PicoPress sensor was positioned (Figure 4). Upon activating the integrated piston within the device, the measurement outcomes were displayed on the PicoPress screen. Results were reported in mmHg in accordance with established medical standards.

## 3. Results and Discussion

### 3.1. Results of Surface Roughness Testing

**Figure 5:**
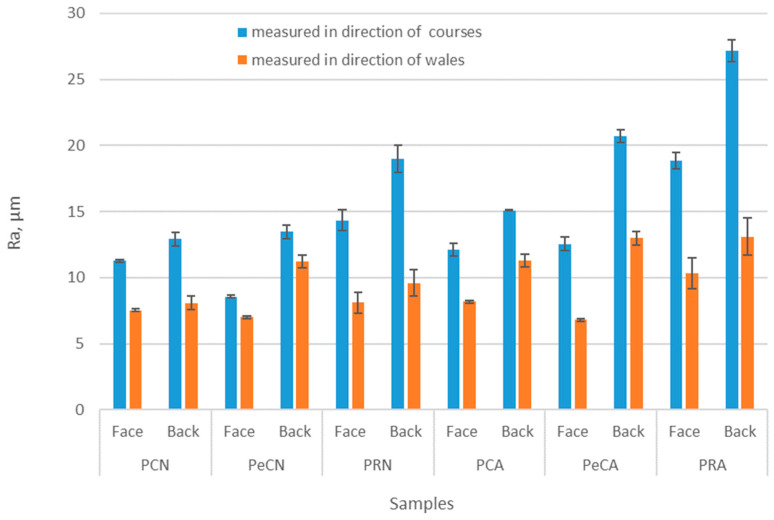
Results of the roughness test—Ra values given for the measurements in the directions of courses and wales, measured on the face and back of the material.

**Figure 6:**
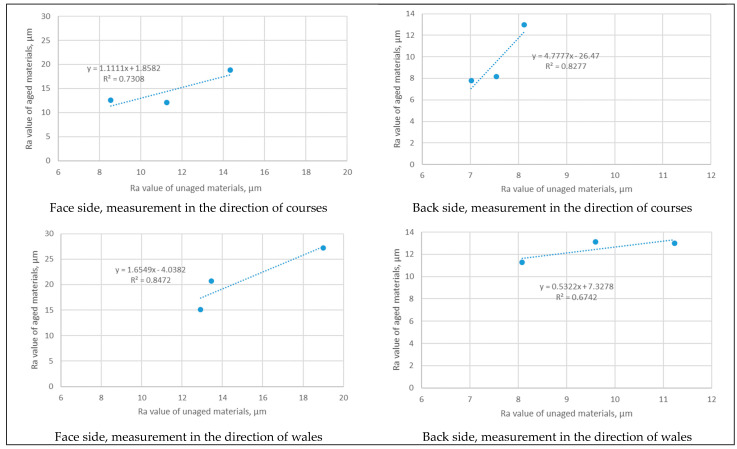
Correlations for Ra values of unaged and aged materials for the face/back side of the material and directions of courses/wales.

**Figure 7:**
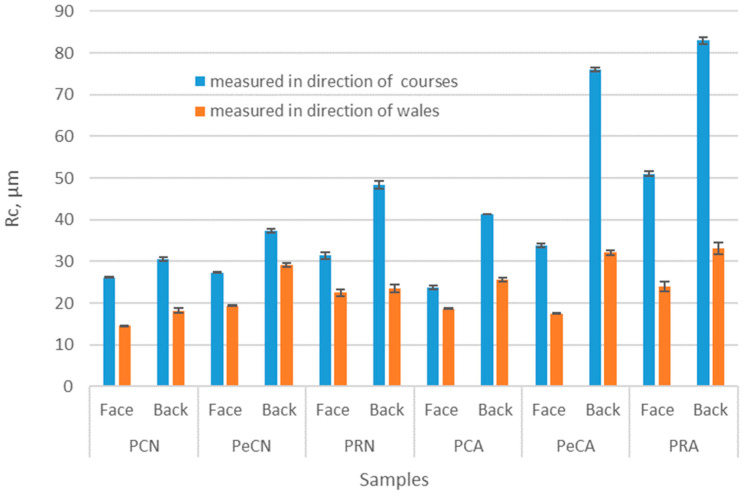
Results of the roughness test—Rc values for the face/back side of the material and directions of courses/wales.

Figure 5, Figure 6 and Figure 7 show the results of the surface roughness testing, more specifically, the values of the parameters Ra and Rc. The measurements were given for the face and the back of the material, as well as for the measurements in the directions of courses and wales. As can be seen, the Ra values of tested materials are in the range of 8.55 to 27.17 μm (Figure 5). The Ra values of materials indicated the differences in surface roughness caused by the use of different polymer yarns for material production. More precisely, it can be seen that the material produced of 100% conventional polyester yarn exhibits higher surface roughness on the face of the material than the same material when the elastane component is added to its structure. This decrease is up to 30% (Figure 6). In contrast, the use of recycled polymer yarn increases the surface roughness in comparison to the use of conventional polymer yarns. Due to this high difference in values, the changes in surface roughness may be expected to be negatively perceived by the wearer, especially when noting that, among a variety of material properties (for example, thickness, elasticity, softness), the surface roughness was confirmed in a previous study to be the most recognizable by the evaluators [10]. 

Regarding the significance of measurement procedures, investigators reported a noticeable difference that was observed in surface roughness between the front and back surfaces of a fabric [2]. As seen in Figure 5, the Ra values of investigated materials concur perfectly with previous findings, as the values for observed samples are higher for the back side of the material. The difference between the two sides of materials is especially pronounced for the material produced of recycled polyester (i.e., samples PRN and PRA). 

As far as the Ra values are observed, the results have pointed out the importance of the definition of measuring procedure. Namely, it can be seen that the Ra values are lower when the measurement is performed in the wales direction (Figure 5). The reason for such a difference between the results of measurements in different directions is to be found in the specific structure of the basic units that form the knitted fabric. If the measurement is taken vertically (i.e., in the direction of wales), the device’s sensor follows the direction of loop wales. In this direction, the connection between the loops is more uniform, resulting in a lower surface roughness. These results differ significantly from previous results reported in the literature [27], where the roughness results showed some differences due to the measurements in different directions, but they were not statistically significant (i.e., the *p*-value was >0.05 in all cases). The reasons for this discrepancy may lie in the nature of the material examined or in insufficiently accurate positioning of the testing instrument in relation to the direction of the basic units of the structure of knitted fabrics. The results of the paired *t*-test for the roughness in the direction of courses vs. the direction of wales (Table 2) suggest that there is a statistically significant difference in roughness between unaged and aged materials for both measurement directions. This conclusion is supported by both the one-tailed and two-tailed tests, with the *p*-values for the direction of the courses (*p* = 0.0057; *p* = 0.0114) and the direction of the wales (*p* = 0.0131; *p* = 0.0262) indicating significance at typical levels, Table 2. 

The results of the parameter Ra show that the surface roughness of conventional polyester fabric increased by up to 7% on the face of the material and by 16% on the back side after aging. This substantiates previous findings in the literature [7] reported about changes in roughness of aged conventional polyester fabrics. Compared to the aged conventional polyester material, the aged polyester material with elastane and the aged recycled material show an increase in roughness. This increase is up to 46% on the face of the material and up to 53% on the back side, making both materials less suitable for prolonged use. The observed increase in material roughness of these two aged materials may contribute to an increase in friction between the material and the wearer’s skin in practice, which in turn affects the increase in the feeling of discomfort.

The experiment presented in this study has so far been small in scale in terms of the number of samples examined. The reason for this is the fact that the natural aging of materials limits the amount of materials that can be exposed to the same environmental factors at the same time. To ensure the accuracy of the measurement data, we used newly calibrated equipment, applied a standardized measurement procedure, and implemented quality control after each set of measurements. The comparison of the Ra values of unaged and aged materials for both fabric sides (i.e., face and back) and test directions of testing (courses and wales), as well as for the corresponding models, are shown in Figure 6. As can be seen, the R^2^ values are higher than 0.6742 in all cases presented. These values indicate a strong to very strong correlation between the roughness results obtained for the investigated materials under specific measurement conditions. A high R^2^; value, typically close to 1, indicates a good fit between the model and the observed data, suggesting that the model predictions are reliable and that there is a strong correlation between the variables being studied.

The results for the mean height of the profile elements Rc show that these values are much lower if the measurement is carried out in the wales direction (Figure 7). Since the knitted fabric has a specific structure consisting of loops, the sensor tracks the loop direction when the measurement is made vertically. As the face and back sides of the knitted fabric are not structurally the same, these differences can also be seen in the graph. The back side shows lower values than the face side. When analyzing the relationship between unaged and aged materials, it can be seen that aged materials have a higher Rc value. This increase can be explained by the separation of the fibers from the yarn structure that occurs during the aging of the material. This increase is lower for the conventional 100% polyester material than for the other tested materials. The increase in the Rc value on the back side is not as pronounced.

With the transition of the engineering process to a consumer-oriented model, efforts are focused on creating desirable products, which include improving the consumer experience in terms of sensory elements [7]. Concerning the relevance of the roughness data for the end user, it should be noted that among the materials examined, conventional polyester fabric is considered to be more favorable for purchase and extended use than polyester fabric with elastane and recycled polyester fabric. This problem is particularly important for certain groups of textile materials that are in direct contact with the skin, such as the sports materials that are the subject of this study. Indeed, increasing the roughness of the fabric has a strong impact on its suitability for a particular application, as rougher material can cause redness on sensitive skin, which can lead to itching, rashes, or exacerbation of conditions such as eczema.

### 3.2. Results of Abrasion Resistance

**Figure 8:**
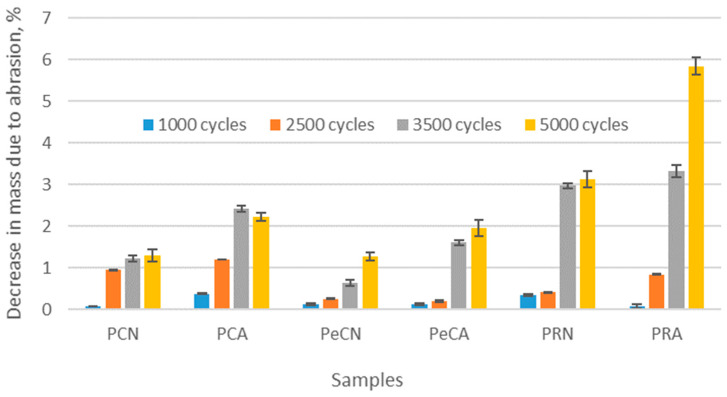
The decrease in material mass due to abrasion after a defined number of abrasion cycles (1000, 2500, 3500, and 5000).

The results of the abrasion resistance of the materials are shown in Figure 8. The results are expressed as percentages of the decrease in mass of the material due to abrasion after a certain number of abrasion cycles (1000, 2500, 3500, and 5000 abrasion cycles). 

The decrease in mass due to abrasion in the unaged polyester materials is between 0.06% and 2.41%, depending on the number of abrasion cycles (Figure 8). The reason for such relatively low values of mass decrease is to be explained by the high tenacity of the conventional polyester fibers. These results are consistent with the conclusions of the previously conducted study on the abrasion resistance of materials made of different fibers [9]. As far as the influence of the elastane component is concerned, the decrease in mass for the elastane-plated material after 1000 and 2500 abrasion cycles is lower than the one for non-plated material. This can be explained by the higher extension of the elastane in the elastane-plated structure. However, further abrasion of unaged materials leads to a higher mass loss of the material plated with elastane yarn, so that the mass loss after 5000 abrasion cycles is very similar to the one of the material made from conventional polyester (precisely, the mass loss for PCN is 1.29 g, while the one for PeCN is 1.27 g). This leads to the conclusion that the influence of the elastane component on the increased abrasion resistance of the textile material does exist but is not as pronounced during prolonged abrasion. This partially supports the result of the study by Tomljenović et al. [10], in which it was shown that a higher addition of elastane to the fiber blend requires a higher number of abrasion cycles for material thinning than is the case with a lower elastane content, i.e., the addition of elastane makes the material more abrasion-resistant. Unlike observed slight differences between conventional materials with and without elastane, the mass loss due to abrasion for the material produced of recycled polyester yarn is rather high (Figure 8). The values of mass loss for the unaged material made of recycled yarn are up to 3.13% and are consistently higher than those of materials produced of conventional polyester (with and without elastane) at each number of abrading cycles. This was to be expected, as this type of raw material was used earlier (i.e., before the recycling process), and the tenacity of its fibers is expected to be lower than that of conventional polyester fibers. Furthermore, the reason for this result could also be found in the lower compactness of the structure of the recycled yarn. This conclusion is in line with the conclusion of the study by Kilic et al. [9]. The study concluded that abrasion triggers a significant loss of material strength and that mass loss is related to the tenacity of the fibers. To sum up, the results of the abrasion suggest that of the three types of polymer materials used, the recycled polyester materials have the lowest abrasion resistance without being exposed to additional aging factors. 

Regarding the difference between aged materials and their unaged counterparts, the results confirm the expected increase in mass loss due to aging. For the materials produced by conventional polyester with and without elastane components, the differences between the mass loss of aged and unaged samples are less pronounced. In the unaged state, the decrease in mass ranges from about 0.12% to 1.26%, while in the aged state, it ranges from about 0.12% to 2.42%. This indicates that aging has a minimal effect on the abrasion resistance of the conventional polyester material, as well as on the elastane blend, compared to the unaged counterparts. The aged recycled polyester also shows a higher loss in mass than its unaged counterpart. The decrease in the mass of the material ranges from approximately 0.21% to 5.84% for aged recycled polyester, compared to approximately 0.27% to 3.13% for unaged recycled polyester. As far as the behavior of the recycled material is concerned, the differences in mass loss observed between this material and materials made of conventional polyester in an unaged state are even more pronounced after the materials have aged. After the highest number of abrasion cycles (in this case, 5000 cycles), the decrease in mass of aged conventional polyester materials is 1.95–2.21%. At the same time, the decrease in mass of the aged recycled polyester material is as high as 5.83%. It is also interesting to note that the material made from recycled polyester exhibits a very sudden mass loss after each abrasion cycle performed. Specifically, the mass loss after 3500 cycles is 3.31%, while after 5000 cycles it is 5.83%. It is to be expected that this trend will continue if the number of abrasion cycles is increased further, thus significantly affecting the quality of the material for further use. In summary, the data show that the aging process carried out on materials made from conventional polyester yarns has led to a slight decrease in mass, with the elastane plating showing no significant effect, while the abrasion resistance of recycled materials has deteriorated significantly. The results of the measurements, which confirm that materials produced using different technologies show very different behavior when exposed to aging, are consistent with the conclusions of the study on the abrasion of knitted fabrics exposed to aging through normal use and washing [12].

The results of the *t*-test comparing the abrasion resistance at the highest number of abrasion cycles between unaged and aged materials are shown in Table 3. As can be seen from Table 3, the Pearson correlation coefficient between the abrasion resistance of unaged and aged materials is 0.9876, indicating a strong positive linear relationship between the two observed variables. Furthermore, both the one-tailed and two-tailed *p*-values are lower than the significance level of 0.05, suggesting that there is a significant difference in the mean abrasion resistance between the observed variables. 

From the user’s point of view, the results of abrasion tests have indicated that the investigated recycled polyester material has a rather low abrasion resistance. It is, therefore, to be expected that the visual appearance of the material surface may deteriorate after a relatively short period of wear. In addition, the durability of such a material is rapidly reduced compared to materials made from conventional polyester, which can have a negative impact on athletes’ performance.

### 3.3. Results of Tensile Testing

**Figure 9:**
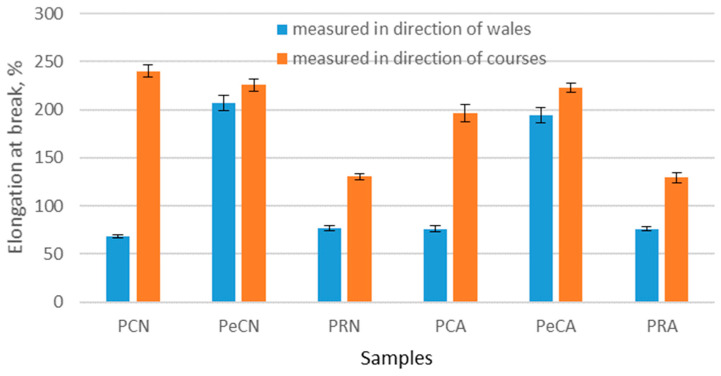
The elongation at break of the materials before and after the aging process measured in the directions of courses and wales.

**Figure 10:**
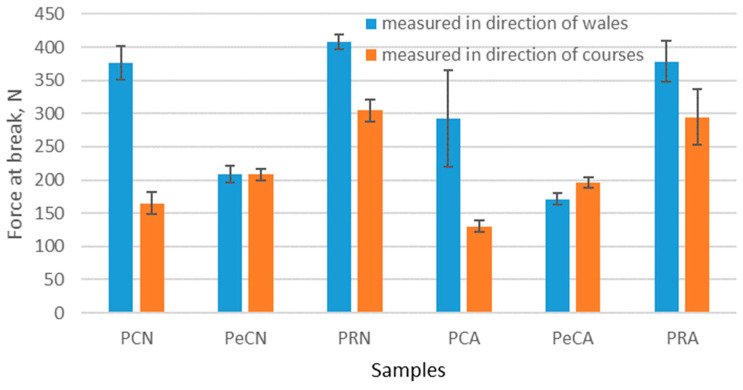
The force at break of the materials before and after the aging process, measured in the directions of courses and wales.

The results of the force at break and the elongation at break of the materials tested in the wales and courses directions, before and after the aging process, are shown in Figure 9 and Figure 10. From Figure 9, it can be seen that the elongation at break before exposure to aging is in the range of 68.23–239.90% and is higher in the course direction for all materials. The difference in the elongation at break is particularly visible with 100% polyester material (material marked as PCN). With this material, the elongation at break in the course direction is 2.5 times higher than in the wale direction. This noted difference is in line with a previous study [24], which has shown that there is a significant difference in elongation at break tests in the directions of the courses and wales of polyester materials. In the case of the material to which elastane has been added, the behavior of the material structure changes so that the differences in the values for the different directions of the material are less pronounced. More precisely, the elongation at break for this type of material in the course direction is only 9% higher than in the wale direction.

As can be seen from the results presented in Figure 9, the aging process did not have a higher impact on the elongation at break of materials. The largest decrease is in 100% polyester material, and it amounts to 11% in the wale direction and 18% in the course direction. The smallest reduction is for recycled polyester material, and it is 1% in both directions. The standard deviation for all elongations at break for unaged and aged materials is low, and there are no statistically significant differences between elongations at break. These results are in contrast to the study by Pušić et al., who show that aging has a significant influence on the change in elasticity of the polyester material [31].

The forces at break of the measured materials are higher in the wale direction. Only for material to which elastane has been added is the force at break similar or higher in the course direction. In analyzing the results of the force at break before aging, it can be seen that the force at break decreases after aging for all measured materials. The highest decrease is for 100% polyester material, which is 22.26%. The smallest decrease is for recycled polyester material, and it is 7.33%. There are statistically significant differences between forces at break in the wale direction (*p*-value is 0.048). The force at break in the course direction exhibits the same behavior. The highest decrease is for 100% polyester material, which is 21.4%. There are no statistically significant differences between forces at break in the course direction (*p*-value is 0.073). It should also be noted that the material with added elastane has a lower force at break in the wale direction than the material without elastane. This observation is similar to the results of a previously published paper [27] where it was shown that polymer material with added elastane exhibits lower force at break than the one without elastane.

**Figure 11:**
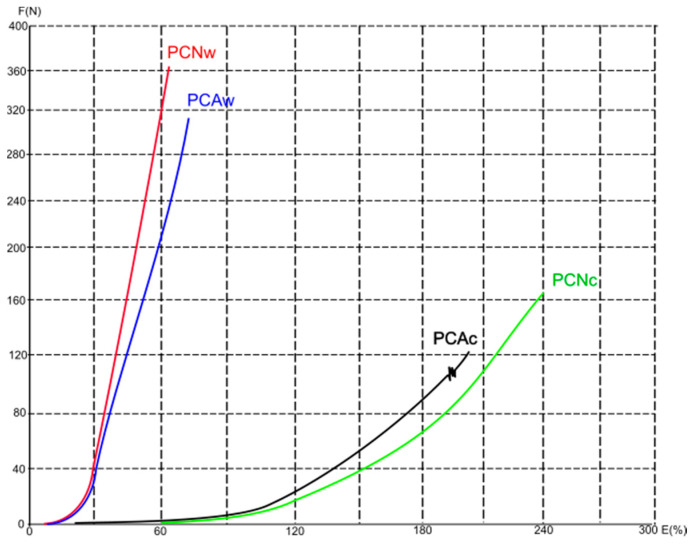
The average force/elongation diagrams for the conventional 100% polyester materials in the wales (w) and course (c) directions.

**Figure 12:**
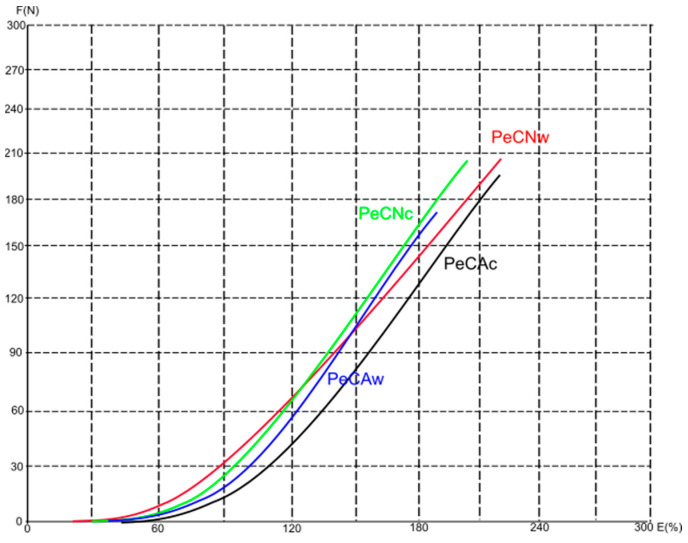
The average force/elongation diagrams for the 87% polyester + 13% elastane materials in the wales (w) and course (c) directions.

**Figure 13:**
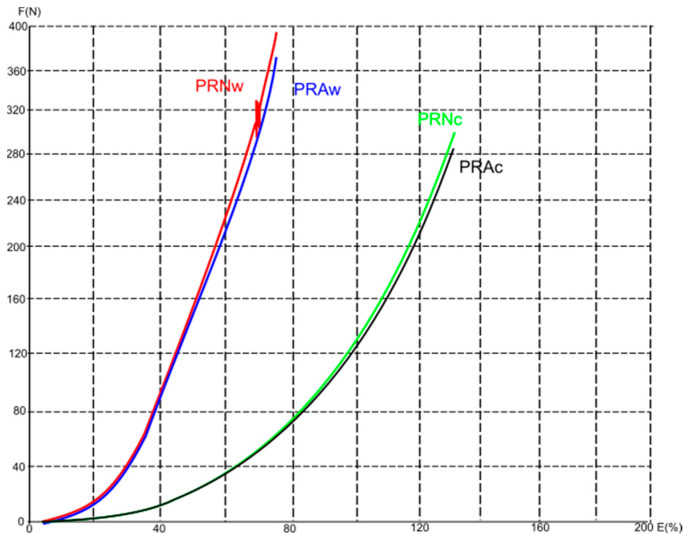
The average force/elongation diagrams for the recycled 100% polyester materials in the wales (w) and course (c) directions.

Figure 11, Figure 12 and Figure 13 show average force/elongation diagrams for the tested materials in the wales (w) and courses (c) directions. Regarding the diagrams for the conventional polyester material and the direction of wales (Figure 11), the PCNw curve shows a rapid increase in force at low elongation, indicating a material with high stiffness and low elongation before breaking. The curve for the aged material measured in the direction of wales (i.e., the PCAw curve) also shows a rapid increase in force, but not as steep as the PCNw curve. As far as the curves for the direction of courses are observed, both the unaged and aged materials show a gradual increase in force with elongation, indicating that the material has moderate stiffness and higher strain in the direction of the courses. Similar behavior is distinguished for the recycled materials with higher overlapping of curves for each observed direction (i.e., the overlap of PRNw and PRAw; PRNc and PRAc), Figure 13. Unlikely, the curves describing the elastane-plated material show a similar trend regardless of measurement direction and aging process, all with a gradual increase in force with elongation, Figure 12.

### 3.4. Results of Bursting Force Testing

**Figure 14:**
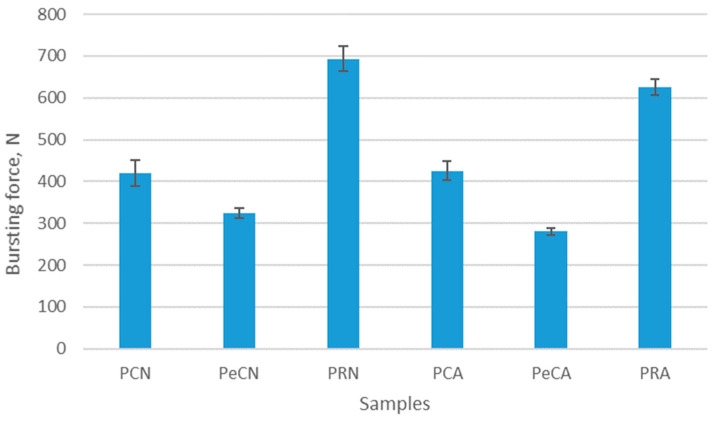
The bursting force of the materials before and after the aging process.

**Figure 15:**
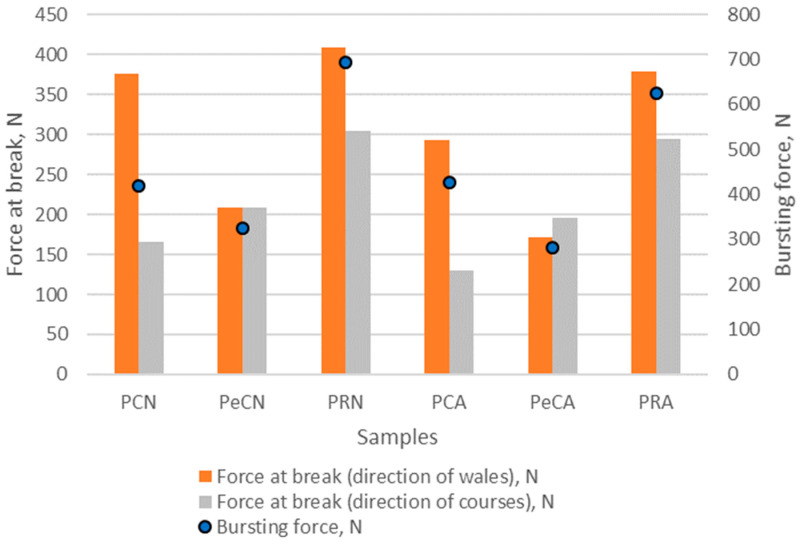
The comparison of bursting force with force at break measured in the directions of wales and courses for unaged and aged materials.

The results of the bursting force testing are shown in Figure 14, while the comparison of bursting force with force at break measured in the directions of wales and courses for unaged and aged materials is shown in Figure 15.

Figure 14 shows that the bursting force of the materials before aging is in the range of 324.58–693.34 N, and after aging, in the range of 280.57–625.88 N. Among investigated materials, the material with added elastane has the lowest bursting force, both in the unaged and aged states. The lower standard deviation for materials with elastane indicates more consistent results. As can be seen, recycled polyester has the highest bursting force among investigated materials. Since sportswear is exposed to a variety of different physical stresses during activities, a high bursting strength ensures that the fabric can withstand these forces without rupturing. From this point of view, recycled polyester material could, therefore, have an advantage over conventional polyester materials (both with and without elastane). Aging reduces the bursting force for all materials, with the exception of the 100% polyester material, where the bursting force increases slightly for 1%. For the material to which elastane was added, the bursting force decreased by 13.5%. Although the bursting force of recycled materials decreases by 9.7% due to aging, it is still much higher than that of conventional materials. The observed advantage of recycled material is, therefore, not reduced even after aging. 

The comparison of the bursting force with the force at break (Figure 15) shows that the recycled polyester materials consistently have the highest values in both directions, making them the strongest fabric of those tested. It is, therefore, suitable for applications that require high strength and durability. Material made from 100% polyester has a moderate bursting force and a force at break values. There is a noticeable decrease in the force at break after aging only, especially in the direction of wales. This indicates that while bursting force is maintained, directional strength decreases with aging. Polyester materials with elastane have the lowest values of bursting force and force at break, indicating that the addition of elastane reduces the overall strength of the fabric. Aging affects all fabrics but in different ways. Recycled polyester retains most of its strength after aging, both in terms of bursting force and force at break, which indicates good aging resistance. Conventional polyester shows a slight increase in bursting force after aging but a significant decrease in the force at break, indicating a loss of directional strength. Polyester with elastane shows a decrease in both the bursting force and the force at break values, indicating that the aging process has a negative effect on the overall and directional strength of the fabric. In summary, materials produced from recycled polyester seem to be best suited for high-strength applications where both overall and directional strength are crucial, and durability over time is important.

### 3.5. Results of Compression Testing

**Figure 16:**
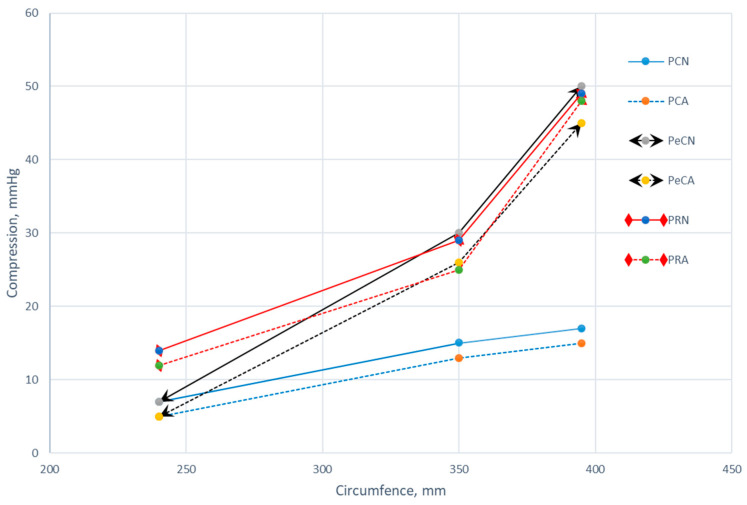
Compression of unaged and aged materials for a different circumference of a cylinder (240 mm, 350 mm, and 395 mm).

Understanding the compression characteristics of polymer materials is crucial for various applications, especially sportswear, because the ability of a material to provide adequate compression can influence its performance and athletes’ comfort. The results of measurement are presented in Figure 16 and Table 4.

The results presented in Figure 16 indicate a noticeable difference in the compression performance of investigated polymer materials based on the material composition (PES conventional, PES + elastane, PES recycled). In particular, the blend of conventional polyester and elastane exhibited higher compression values than conventional polyester under comparable conditions. More specifically, the compression results of unaged 100% polyester material are 7–17 mmHg, while for the polyester with elastane, it is 7–50 mmHg. This indicates that the presence of elastane in the material structure has an important influence on increasing the compressibility of the material. This behavior can be explained by the fact that the introduction of elastane yarns into the course of the fabric influences the longitudinal and transverse shrinkage of the structure. Therefore, the elastane-plated knitted fabric has a fuller structure and more loops per unit area than the non-plated knitted fabric, which has an effect on increasing the compression values. This observation is in good agreement with the results of the study comparing fully and partially plated knitted fabrics [15]. The aforementioned study showed that at different circumferences and sinking depths, the fully plated elastane fabric still has higher compression values than the partially plated materials. Thus, this led to a similar conclusion that the elastane component has a positive effect on increasing the compression properties of the knitted fabric. The study did not include a comparison of non-plated and plated knitted fabrics.

In all the material compositions examined, the aged specimens consistently exhibited lower compression values compared to their unaged counterparts. More specifically, the material compression values for the unaged materials ranged from 7 to 50 mmHg, while the values for the aged materials ranged from 5 to48 mmHg. The *t*-test was performed to gain further insight into the compression results between unaged materials (variable 1) and aged materials (variable 2). As shown in Table 4, the Pearson correlation coefficient between the two variables is 0.9972. This Pearson coefficient value indicates a very strong and positive linear relationship between the observed variables. In addition, the t-statistic is 6.0474, which is much larger than the critical t-value for both the one-tailed test (equals 1.8596) and the two-tailed test (equals 2.3060). This also confirms the statistical significance of the difference between the means of the two observed variables. Moreover, both *p*-values (one-tailed and two-tailed) are lower than the significance level of 0.05, which leads us to conclude that there is a significant difference in the mean compression values between the unaged and aged material groups. These results indicate that the structural integrity of the polymeric materials studied may deteriorate over time, resulting in reduced compression performance. As expected, the results presented have also shown that the circumference of the cylinder had a notable impact on the compression of materials. Namely, as the circumference increased, the compression values generally tended to rise. This suggests that larger areas require higher compression levels to achieve similar effects, which aligns with the basic principles of material mechanics.

## 4. Conclusions 

The intention of this study was to observe the changes in polyester material properties due to aging, which may have a negative impact on an athlete’s performance. The results showed that the abrasion resistance of recycled material had deteriorated significantly due to aging. Both the conventional polyester fabric with elastane and the recycled polyester fabric have a higher roughness on the face and back of the material. These materials also have higher compression values compared to conventional polyester materials. When compared to conventional materials, recycled polyester material retains less elasticity after aging but has a higher resistance to the effects of force up to breakage. In summary, while the use of recycled polyester yarn for material production has some advantages, such as higher resistance to breaking forces, conventional polyester yarn is still more favorable overall due to better abrasion resistance, lower surface roughness, and better retention of elasticity after aging. This makes conventional polyester fabric more suitable for extended use by professional football players.

The study has highlighted the differences in the properties of polyester materials due to aging. In today’s competitive world where athletes’ performance is paramount, the results of the study should be used to design even more functional materials that meet the needs of users. Future research in this field should focus on the further development of sustainable and eco-friendly polymer materials that can maintain their properties over time to a greater extent than the recycled polyester material studied in this research.

## Figures and Tables

**Table 1:** Material designation and description.

Material Designation	Composition	Yarn Type	Aging
PCN	100% polyester	conventional yarn	unaged
PeCN	87% polyester + 13% elastane	conventional yarn	unaged
PRN	100% polyester	recycled yarn	unaged
PCA	100% polyester	conventional yarn	aged
PeCA	87% polyester + 13% elastane	conventional yarn	aged
PRA	100% polyester	recycled yarn	aged

**Table 2:** The *t*-test for the results of material roughness (variables: roughness of unaged materials, roughness of aged materials) measured in the directions of courses and wales.

	Direction of Courses	Direction of Wales
Mean	13.2533	8.5983
Variance	12.0555	2.4075
Observations	6	6
Pearson Correlation	0.9277	0.8631
Hypothesized Mean Difference	0	0
df	5	5
t Stat	−3.8961	−3.1228
P(T ≤ t) one-tail	0.0057	0.0131
t Critical one-tail	2.0150	2.0150
P(T ≤ t) two-tail	0.0114	0.0262
t Critical two-tail	2.5706	2.5706

**Table 3:** The *t*-test for the results of material abrasion.

*t*-Test: Paired Two Sample for Means	
Pearson Correlation	0.9876
Hypothesized Mean Difference	0
df	5
t Stat	3.9908
P(T ≤ t) one-tail	0.0052
t Critical one-tail	2.0150
P(T ≤ t) two-tail	0.0104
t Critical two-tail	2.5706

**Table 4:** The *t*-test for the results of material compression.

*t*-Test: Paired Two Sample for Means	
Pearson Correlation	0.9972
Hypothesized Mean Difference	0
df	8
t Stat	6.0474
P(T ≤ t) one-tail	0.0002
t Critical one-tail	1.8596
P(T ≤ t) two-tail	0.0003
t Critical two-tail	2.3060

## Data Availability

The original contributions presented in the study are included in the article, further inquiries can be directed to the corresponding author.

## References

[1] Militky J., Mazal M. (2007). Image Analysis Method of Surface Roughness Evaluation. Int. J. Cloth. Sci. Technol..

[2] Mooneghi S.A., Saharkhiz S., Varkiani S.M.H. (2014). Surface Roughness Evaluation of Textile Fabrics: A Literature Review. J. Eng. Fiber. Fabrics.

[3] Salopek Čubrić I., Čubrić G., Majumdar A. (2023). Sensory attributes of knitted fabrics intended for next-to-skin clothing. J. Text. Inst..

[4] Čubrić I.S., Čubrić G., Perry P. (2019). Assessment of Knitted Fabric Smoothness and Softness Based on Paired Comparison. Fibers Polym..

[5] Chae Y. (2022). Color appearance shifts depending on surface roughness, illuminants, and physical colors. Sci. Rep..

[6] Lee S. (2022). Study of superhydrophobicity according to surface structure of knitted fabrics. Text. Res. J..

[7] Šaravanja A., Dekanić T., Pušić T., Kaurin T. Primary sensory indices of polyester fabric exposed to aging. Proceedings of the 5th Scientific–Professional Symposium TZG 2023.

[8] Mikučioniene D., Čepukone L., Milašiene D. (2018). Investigation on mechanical and thermal properties of knits from peat fibers and their combination with other natural fibers. Text. Res. J..

[9] Balci Kilic G., Okur A. (2019). Effect of yarn characteristics on surface properties of knitted fabrics. Text. Res. J..

[10] Tomljenović A., Skenderi Z., Kraljević I. Evaluation of usage quality and thermal comfort of male socks. Proceedings of the 8th International Textile Conference.

[11] Gupta S.K., Goswami K.K., Majumdar A. (2018). Optimization of durability of Persian hand-knotted wool carpets by using desirability functions. Text. Res. J..

[12] Jabbar A., Palacios-Marín A.V., Ghanbarzadeh A., Yang D., Tausif M. (2023). Impact of conventional and modified ring-spun yarn structures on the generation and release of fragmented fibers (microfibers) during abrasive wear and laundering. Text. Res. J..

[13] Shi Q.Q., Jiao J., Shin K., Chow H.K., Lau N. (2023). Study of cyclists’ skin deformation for compression skinsuit design. Text. Res. J..

[14] Hakala T., Puolakka A., Nousiainen P., Vuorela T., Vanhala J. (2018). Application of air bladders for medical compression hosieries. Text. Res. J..

[15] Lozo M., Lovričević I., Pavlović Ž., Vrljičak Z. (2021). Designing compression of preventive compression stockings. J. Eng. Fiber. Fabrics.

[16] Liu T., Gao Y., Fan W., Gao X., Ma J. (2022). Predictions of the axial tensile property of the unidirectional composite influenced by microfiber breakage defects. Text. Res. J..

[17] Xie Z., Li J., Liu Y., Liu K., Li W. (2023). Effect of structural parameters on tensile properties of alumina fabrics. Text. Res. J..

[18] Shi Y., Liu R., Wong C., Ye C., Lv J. (2024). Prediction of tensile behavior of compression therapeutic biomedical materials by mesoscale laid-in loop model. Polymer.

[19] Huang Y., Song X. (2023). Study on an innovative knitting technology of spacer fabrics and the low-velocity impact properties of its composites. Text. Res. J..

[20] Li X., Jiang G., Nie X., Ma P., Gao Z. (2015). Knitting Technologies And Tensile Properties Of A Novel Curved Flat-Knitted Three-Dimensional Spacer Fabrics. Autex Res. J..

[21] Cionek C.A., Nunes C., Freitas A., Homem N., Muniz E., Amorim T. (2021). Degradation study of polyester fiber in swimming pool water. Text. Res. J..

[22] Sørensen L., Groven A.S., Hovsbakken I.-A., Del Puerto O., Krause D.F., Sarno A., Booth A.M. (2021). UV degradation of natural and synthetic microfibers causes fragmentation and release of polymer degradation products and chemical additives. Sci. Total Environ..

[23] Arhant M., Le Gall M., Le Gac P.Y., Davies P.D. (2019). Impact of hydrolytic degradation on mechanical properties of PET—Towards an understanding of microplastics formation. Polym. Degrad. Stab..

[24] Petrov A., Salopek Čubrić I., Čubrić G. (2024). Influence of Aging on the Physical Properties of Knitted Polymeric Materials. Polymers.

[25] Salopek Čubrić I., Čubrić G., Katić Križmančić I., Kovačević M. (2022). Evaluation of Changes in Polymer Material Properties Due to Aging in Different Environments. Polymers.

[26] Salopek Čubrić I., Čubrić G., Potočić Matković V.M. (2021). Behavior of Polymer Materials Exposed to Aging in the Swimming Pool: Focus on Properties That Assure Comfort and Durability. Polymers.

[27] Katić Križmančić I., Salopek Čubrić I., Potočić Matković V.M., Čubrić G. (2023). Changes in Mechanical Properties of Fabrics Made of Standard and Recycled Polyester Yarns Due to Aging. Polymers.

[28] (1996). Geometrical Product Specifications (GPS)—Surface Texture: Profile Method—Nominal Characteristics of Contact (stylus) Instruments.

[29] (2013). Textiles—Tensile Properties of Fabrics—Part 1: Determination of Maximum Force and Elongation at Maximum Force Using the Strip Method.

[30] (2020). Textile Ball Burst Testing.

[31] Pušić T., Vojnović B., Flinčec Grgac S., Čurlin M., Malinar R. (2023). Particle Shedding from Cotton and Cotton-Polyester Fabrics in the Dry State and in Washes. Polymers.

